# Mixed-Beam Approach for High-Risk Prostate Cancer Carbon-Ion Boost Followed by Photon Intensity-Modulated Radiotherapy: Preliminary Results of Phase II Trial AIRC-IG-14300

**DOI:** 10.3389/fonc.2021.778729

**Published:** 2021-11-17

**Authors:** Giulia Marvaso, Barbara Vischioni, Matteo Pepa, Mattia Zaffaroni, Stefania Volpe, Filippo Patti, Federica Bellerba, Sara Gandini, Stefania Comi, Giulia Corrao, Dario Zerini, Matteo Augugliaro, Cristiana Fodor, Stefania Russo, Silvia Molinelli, Mario Ciocca, Rosalinda Ricotti, Francesca Valvo, Tommaso Giandini, Barbara Avuzzi, Riccardo Valdagni, Ottavio De Cobelli, Federica Cattani, Ester Orlandi, Barbara Alicja Jereczek-Fossa, Roberto Orecchia

**Affiliations:** ^1^ Division of Radiotherapy, Istituto Europeo di Oncologia (IEO), European Institute of Oncology Istituto di Ricovero e Cura a Carattere Scientifico (IRCCS), Milan, Italy; ^2^ Department of Oncology and Hemato-Oncology, University of Milan, Milan, Italy; ^3^ Clinical Department, National Center for Oncological Hadrontherapy (CNAO), Pavia, Italy; ^4^ Department of Experimental Oncology, Istituto Europeo di Oncologia (IEO), European Institute of Oncology Istituto di Ricovero e Cura a Carattere Scientifico (IRCCS), Milan, Italy; ^5^ Medical Physics Unit, Istituto Europeo di Oncologia (IEO), European Institute of Oncology Istituto di Ricovero e Cura a Carattere Scientifico (IRCCS), Milan, Italy; ^6^ Medical Physics Unit, Fondazione Istituto di Ricovero e Cura a Carattere Scientifico (IRCCS) Istituto Nazionale dei Tumori, Milan, Italy; ^7^ Department of Radiation Oncology 1, Fondazione Istituto di Ricovero e Cura a Carattere Scientifico (IRCCS) Istituto Nazionale dei Tumori, Milan, Italy; ^8^ Division of Urology, Istituto Europeo di Oncologia (IEO), European Institute of Oncology Istituto di Ricovero e Cura a Carattere Scientifico (IRCCS), Milan, Italy; ^9^ Scientific Directorate, Istituto Europeo di Oncologia (IEO), European Institute of Oncology Istituto di Ricovero e Cura a Carattere Scientifico (IRCCS), Milan, Italy

**Keywords:** carbon-ion radiotherapy, intensity modulated radiotherapy, high-risk prostate cancer, phase II study, mixed-beam approach

## Abstract

**Purpose:**

This study represents a descriptive analysis of preliminary results of a Phase II trial on a novel mixed beam radiotherapy (RT) approach, consisting of carbon ions RT (CIRT) followed by intensity-modulated photon RT, in combination with hormonal therapy, for high-risk prostate cancer (HR PCa) with a special focus on acute toxicity.

**Methods:**

Primary endpoint was the evaluation of safety in terms of acute toxicity. Secondary endpoints were early and long-term tolerability of treatment, quality of life (QoL), and efficacy. Data on acute and late toxicities were collected according to RTOG/EORTC. QoL of enrolled patients was assessed by IPSS, EORTC QLQ-C30, EORTC QLQ-PR25, and sexual activity by IIEF-5.

**Results:**

Twenty-six patients were enrolled in the study, but only 15 completed so far the RT course and were included. Immediately after CIRT, no patients experienced GI/GU toxicity. At 1 and 3 months from the whole course RT completion, no GI/GU toxicities greater than grade 2 were observed. QoL scores were overall satisfactory.

**Conclusions:**

The feasibility of the proposed mixed treatment schedule was assessed, and an excellent acute toxicity profile was recorded. Such findings instil confidence in the continuation of this mixed approach, with evaluation of long-term tolerability and efficacy.

## Introduction

Prostate cancer (PCa) is the most common solid organ malignancy in men, and radiotherapy (RT) plays a significant role in the treatment of organ-confined or locally advanced disease (1). Although low- and intermediate-risk PCa show excellent outcomes with surgery or RT, high-risk (HR) disease PCa continues to have a high rate of recurrence and progression, both locally and distantly, making research necessary for escalation or combined strategies.

At least 17–31% of these men present with HR localized or locally advanced disease (2) and need a curative treatment, which includes surgery or external beam radiotherapy (EBRT) combined with androgen deprivation therapy (ADT), and an optional brachytherapy boost (3). From a RT perspective, the most peculiar biological feature of PCa is its low α/β ratio, corresponding to a relative radio-resistance, which has fostered the development through the years of different schedules with varying degrees of hypofractionation. Many of these studies (4, 5) have demonstrated to be equally if not more effective in terms of local control and less likely to cause side effects. However, the role of hypofractionation with stereotactic body RT (SBRT) in HR PCa patients remains controversial, especially when it becomes necessary to perform elective pelvic nodal irradiation (6).

In parallel, dose-escalation studies, particularly with a dose boost to the dominant intraprostatic lesions (DIL), have shown an advantage in terms of local control of disease (7, 8), although limited by greater toxicity to adjacent organs such as the bladder and anterior wall of the rectum (9).

In this context, the use of heavy particles was proven to be both safe and effective. In fact, firstly, they allow to reach a steep dose gradient due to the inverted profile of in-depth dose deposition compared to photons, which permits a greater sparing of organs at risk (OARs) (10, 11). Secondly, carbon-ion radiotherapy (CIRT), already been in use in various Centers at an experimental level for more than 10 years, demonstrated a greater efficacy compared to standard radiation techniques due to its peculiar physical features. It is now widely accepted that beams of high linear energy transfer (LET) particles can offer a biological advantage for radioresistant malignancies due to their higher relative biological effectiveness (RBE) (12–14).

In the light of improving outcomes in HR PCa patients without compromising treatment safety, we explored the use of carbon ions to escalate the dose to the prostate and the addition of a standard photon treatment to the pelvic lymph nodes.

In fact, the purpose of this prospective phase II study, sponsored by the Italian Association of Cancer Research (Associazione Italiana per la Ricerca sul Cancro, AIRC), is to evaluate the feasibility of a radiation schedule that comprises a dose boost to the prostate, delivered with carbon ions, followed by a conventional course of pelvic photon RT in patients affected by HR PCa undergoing neoadjuvant and adjuvant long-term hormone therapy.

This study represents a descriptive analysis of preliminary results, with a special focus on acute toxicity. In particular, only acute toxicity events were collected due to the short patients’ follow-up available. The analysis on oncological outcomes and late toxicity events will be performed when more mature data will be collected.

## Materials and Methods

### Trial Characteristics

The protocol was approved by the Ethics Committee (R86/14-IEO98) of the European Institute of Oncology (IEO), coordinating center, and subsequently presented and registered to the ethics committees of the other participating centers, and has been registered at ClinicalTrials.gov (NCT02672449). The study was designed as a prospective, multicentric, phase II open-label trial. The patients have been enrolled at three radiation oncology facilities in northern Italy, namely, National Center of Oncological Hadrontherapy (Centro Nazionale di Adroterapia Oncologica, CNAO) in Pavia, National Cancer Institute (Fondazione IRCCS Istituto Nazionale dei Tumori, INT) in Milan, and European Institute of Oncology IRCCS (Istituto Europeo di Oncologia, IEO) in Milan.

The trial was supposed to enrol 65 consecutive patients (15); however, due to delays in authorizations and to the emergence of competitive surgical trials, it recruited a total of 26 patients. Sample size has been recalculated accordingly, by considering the actual accrual. All enrolled patients signed an informed consent before starting treatment. The study protocol has been previously published (NCT02672449); therefore, patients’ selection, treatment delivery, outcomes of the study, and statistical analyses will be only briefly described hereafter (15).

### Patients Selection

This study included patients affected by HR PCa, as defined by National Comprehensive Cancer Network (NCCN) risk categories [T3a and/or prostate-specific antigen (PSA) >20 ng/ml and/or Gleason score (GS) 8–10]. Inclusion criteria are listed in **Table 1**.

**Table 1:** Inclusion and exclusion criteria.

INCLUSION CRITERIA	EXCLUSION CRITERIA
• Histologically confirmed adenocarcinoma of the prostate, high- risk category according to NCCN version 1.2016 (cT3a and/or PSA >20 ng/mL and/or Gleason score of 8-10)	• Previous pelvic RT
	• Previous prostatectomy
	• Concomitant inflammatory bowel disease or other serious systemic comorbidities
• Age > 18 years	• Previous invasive cancer (within 5 years before the PCa diagnosis unless the patient has been free from disease for at least 3 years) except for nonmelanoma skin malignancies
• cN0 and cM0	
• Eastern Cooperative Oncology Group (ECOG) Performance Status < 2	
• ADT recommended	
○ 3 months before RT	• Presence of hip prosthesis
○ Concomitant to RT	
○ up to 2 years after the end of RT	
• Good urinary flow (peak flow >10 mL/s)	
• Written informed consent	

ECOG, Eastern Cooperative Oncology Group; NCCN, National Comprehensive Cancer Network; PCa, prostate cancer; PSA, prostate-specific antigen; RT, radiotherapy.

### Radiation Therapy Treatment Planning and Delivery

Computed tomography (CT) simulation, volumes of interest contouring, and treatment delivery were performed following the previously described methodology (15, 16).

In particular, all patients first received the CIRT boost to the prostate and to the proximal third of the seminal vesicles at CNAO. The dose prescribed to the planning target volume (PTV) boost was 16.6 Gy (RBE) in four fractions [4.15 Gy (RBE)/fraction, over 1 week]. The CIRT technique used at CNAO (17) consists of two lateral opposed beams, with the PTV receiving at least 95% of the prescribed dose. To complete the RT course, the patients received intensity-modulated RT (IMRT), with the clinical target volume (CTV) including the whole pelvis, and the PTV derived as a 5 mm CTV expansion. Total dose to the PTV pelvis ranged from 45 to 50.4 Gy in 1.8–2 Gy/fraction. Both PTV boost and PTV pelvis received at least 95% of the prescribed dose. Dose constraints to the OARs were derived by considering the plan sum, that is, CIRT + IMRT course. Further details on treatment delivery are available in the study protocol (15).

### Assessment of Quality of Life and Follow−Up

The primary endpoint of this study was to evaluate the feasibility and safety of the proposed treatment through the evaluation of acute side effects by physician reported outcomes, according to the Radiation Therapy Oncology Group (RTOG)/European Organisation for Research and Treatment of Cancer (EORTC) scale. This was achieved by assessing the percentage of patients who report at least one episode of grade (G) 3 or 4 toxicity during treatment or within 1 month after RT. Side effects were also evaluated by patients’ reported outcomes with dedicated questionnaires for treatment-related quality of life (QoL), according to EORTC quality of life-core 30 (QLQ-C30), international prostatic symptoms score (IPSS), and international index of erectile function (IIEF-15) questionnaires. Toxicities evaluated at 3, 12, and 24 months have been considered as secondary endpoints. During follow-up, erectile function was evaluated with the IIEF questionnaire (minimum = 1, serious; maximum = 25, optimal condition). The state of prostatic symptoms was evaluated through the IPSS questionnaire (minimum = 0, no symptoms; maximum score 25 = acute symptoms). The “Global Health Status” based on the EORTC QLQ-C30 questionnaire was described through median and interquartile range. The efficacy of treatment was investigated as secondary endpoint in terms of biochemical response, through prostate specific antigen evaluation every 3 months.

### Statistical Analysis

The primary endpoint of this analysis was acute toxicity that was tested by simply counting the number of patients free from cumulative 1-month acute toxicity after RT.

Categorical variables were reported as frequencies (percentages), whereas continuous variables were summarized with the median value and interquartile range (25th–75th percentiles). We evaluated time trends of IPSS, EORTC QLQ-C30, and the IIEF-5 questionnaires. The missing IPSS scores (n=4) were replaced by the median score at the same time point.

All scales of EORTC QLQ-C30 (functioning scales: Physical Functioning, Role Functioning, Emotional Functioning, Cognitive Functioning, Social Functioning; general health status scales: Global Health Status/QoL; symptom scales: Fatigue, Nausea/Vomiting, Pain, Dyspnoea, Insomnia, Appetite loss, Constipation, Diarrhoea, Financial Problems) were built according to the EORTC manual and transformed to 0–100 scales, with higher scores reflecting either more symptoms or higher levels of functioning or QoL.

For EORTC QLQ-C30 questionnaire, imputation of missing answers was performed as follows: if a patient answered less than half the questions in a scale, the scale was considered to be missing; if a patient answered at least half of the questions in a scale, the average score of the answered questions was calculated and imputed as the response to questions which had not been answered.

For IIEF-15 questionnaire, all observations with more than 30% missing items at a given time point were excluded from the analysis. In all other cases, a missing item was replaced with the mean score of the items from the same domain where available, and with the median value of the item at the same time point when all the items from the same domain were missing.

Within-patient score changes of IPSS, every scale of EORTC QLQ-C30 and IIEF-5 questionnaires were calculated at each time point from baseline. The baseline was defined as the time point right before RT start, meaning 3 months after ADT administration, as specified in the study protocol (15). Linear mixed models for repeated measures were used to detect a trend in the changes. All estimates were adjusted for the baseline score. Residuals from full models were checked to assess normal distribution, and boxplots of the score changes were provided for the main results.

A two-sided p value < 0.05 was considered significant for all statistical analyses.

The analyses were performed using SAS software (SAS Institute Inc., Cary, USA), version 9.4, and R software (https://www.Rproject.org), version 3.5.2.

## Results

### Study Population

Since October 2017, 26 consecutive patients who fulfilled the inclusion criteria have been treated. Sixteen patients have completed the prescribed treatment according to protocol guidelines so far, and one patient dropped out the protocol due to non-PCa-related clinical motivations, so 15 patients were included in the analysis. A follow-up of at least 3 months was available for them. All patients underwent concomitant ADT. Patients’ characteristics are listed in **Table 2**.

**Table 2:** Statistics of patients, tumour, and treatment characteristic*.

VARIABLES	CATEGORIES	STATISTICS
Age, Median (IQR)		74 (59-83)
iPSA, Median (IQR)		12.3 (3.3-63.1)
PSA preRT, median (IQR)		1.2 (0.13-23.57)
T, n (%)	cT1a-c	3 (15)
	cT2a-c	10 (50)
	cT3a	7 (35)
Total GS, n (%)	6 (GG = 1)	1 (5)
	3+4 (GG = 2)	1 (5)
	4+3 (GG = 3)	3 (15)
	8 (GG = 4)	12 (60)
	9 (GG = 5)	3 (15)

GG, grade group; GS, Gleason score; iPSA, initial prostate-specific antigen; IQR, interquartile range; RT, radiotherapy; T, tumour. *Data available for 20 patients.

### Toxicity Outcomes

Overall, patients’ tolerance to treatment was acceptable. After the CIRT boost, no patients experienced gastrointestinal (GI)/genitourinary (GU) toxicity. At 1 and 3 months from RT completion (CIRT followed by IMRT), no GI or GU toxicities greater than grade (G) 2 were observed. In details, considering acute GU toxicity, eight patients have not reported any toxicity. Concerning GI, five patients presented G1 acute toxicity and two of them G2 (**Table 3**). Longer follow-up (12 months) was available for seven patients, with one patient presenting GU toxicity classified as G1 and one patient presenting GU toxicity reported as G2.

**Table 3:** Induced acute and late toxicity.

Variable		Grade	Number of patients (%*)
Acute toxicity	GU	0	8 (53.3%)
		1	5 (33.3%)
	GI	0	12 (80.0%)
		1	1 (6.7%)
Late toxicity^a^	GU	0	5 (53.3%)
		1	0 (0%)
		2	1 (6.7%)
	GI	0	5 (33.3%)
		1	1 (6.7%)

GI, gastrointestinal; GU, genitourinary.

a Missing data for one patient.

*Percentage refers to the whole cohort of patients (15).

### Quality of Life Scores

**Figure 1:**
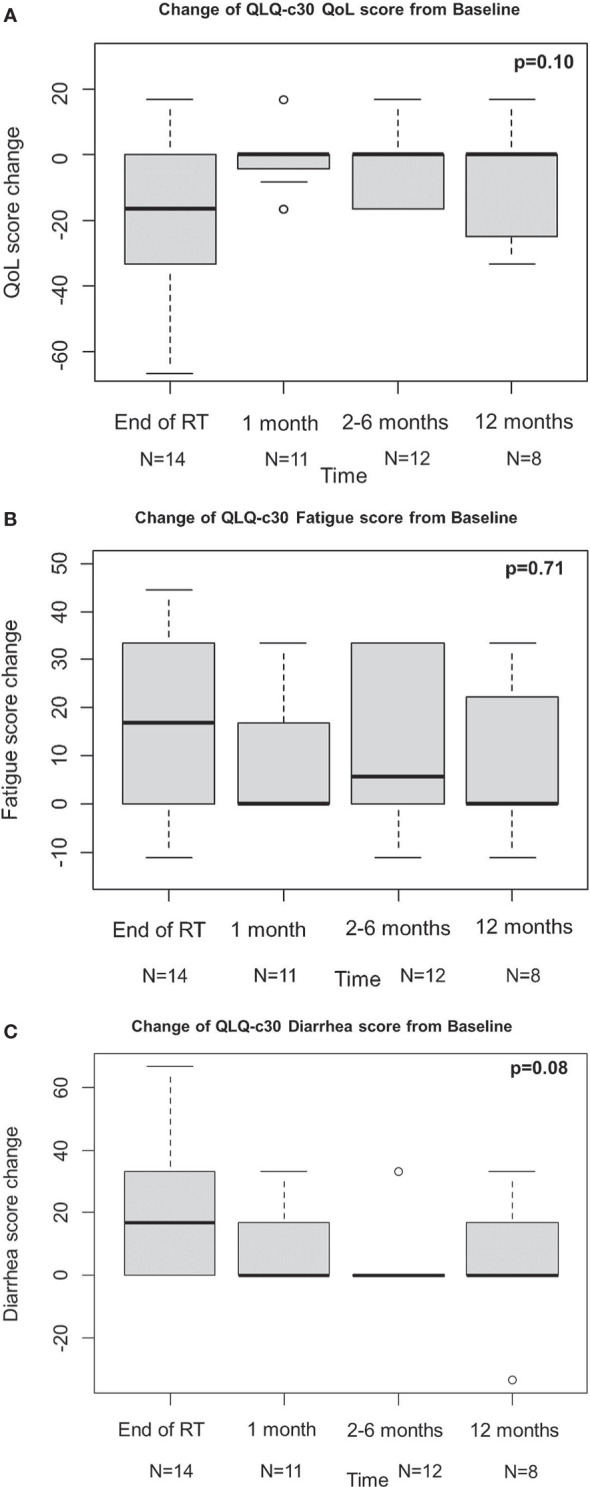
Change of QLQ-c30 QoL score **(A)**, fatigue score **(B)**, diarrhea score **(C)** from baseline.

**Figure 2:**
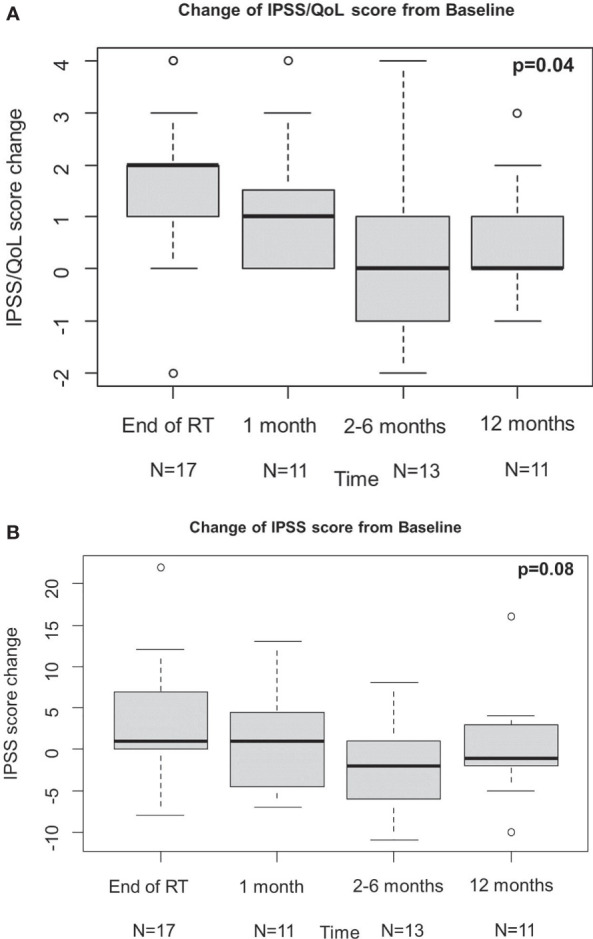
Results of IPSS/QoL **(A)** and IPSS **(B)**. IPSS, international prostatic symptoms score; QoL, quality of life.

**Figure 3:**
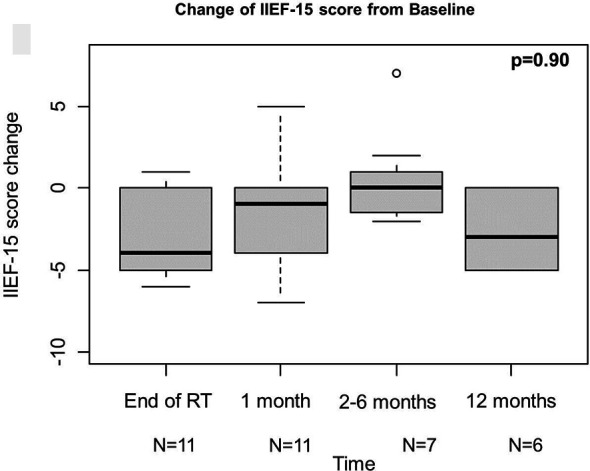
Results of IIEF-15. IIEF-15, international index of erectile function.

QLQ-c30 QoL was analysed to evaluate the urinary function, and revealed an overall improvement from baseline at 1 month, even if not statistically significant, which kept constant at the following time points (**Figure 1A**). These results are consistent with those obtained from IPSS analysis (**Figures 2A, B**). Change of IPSS/QoL score was positive across the considered time points, as a statistically significant marked improvement from baseline was observed (p = 0.04) (**Figure 2A**). Similar findings, despite not statistically significant (p = 0.10), were observed considering the IPSS questionnaire, with no significant deterioration from baseline at all the considered time points. In particular, no IPSS score changes were observed at 1 month after baseline and after RT completion (**Figure 2B**). Considering the QLQ-c30 fatigue score change, a trend towards improvement from baseline was observed, especially at 1 month, even if not statistical significant (p = 0.71) and maintained across the considered time points (**Figure 1B**). The same considerations hold for gastrointestinal diarrhoea, as evaluated by QLQ-c30, with an improvement from baseline (**Figure 1C**). The analysis of IIEF-5 did not show any significant change of erectile function from baseline (p = 0.90), although a worsening of function was observed at the end of RT, which gradually improved at the following time points. A worsening of erectile function was also observed after 12 months, but only six score changes from baseline were available at that time point (**Figure 3**).

### Oncological Outcomes

As stated above, a complete evaluation of oncological outcomes will be object of a separate investigation; however, preliminary results are reported here. As of July 2021, no patients experienced biochemical recurrence. Median PSA value at diagnosis was 12.37 ng/ml [interquartile range (IQR) 8.38–25.00 ng/ml]. After 3 months of hormone therapy and before radiation treatment, median PSA was 1.2 ng/ml (IQR 0.49–5.5 ng/ml). At last follow-up (median 6 months, IQR 3–12 months), median PSA was 0.08 ng/ml (IQR 0.02–0.15 ng/ml).

## Discussion

The present investigation aimed at evaluating preliminary outcomes of a novel mixed-beam approach for HR PCa CIRT followed by photon IMRT on prostate and pelvic lymph nodes, with a focus on acute GI and GU toxicity and QoL. At the current state, the mixed treatment schedule proposed herein shows an optimal 1-month acute toxicity profile. This reflects on the patient-reported QoL scores, which were overall satisfactory, as the changes with respect to baseline values showed an improvement or at least a non-worsening.

The optimal management of locally advanced HR PCa is still a matter of debate, with a rate of recurrence that still remains high (55% at 10 years), even when ADT is administered concomitantly (18, 19). As of today, it is difficult to compare the efficacy of different radical treatment strategies (i.e., surgery, RT, new agents added to standard ADT and RT) in HR PCa, due to the scarcity of randomized controlled trials. Among the most recent experiences that are currently investigating the use of second-generation ADT in combination with local approaches, results are awaited from STAMPEDE (abiraterone) (20), ENZARAD (NCT02446444, enzalutamide), and ATLAS (21), ARNEO (22), PROTEUS (NCT03767244, apalutamide) trials.

Modern RT, including IMRT and hypofractionation, has a central role among the available treatment options. However, currently, there is no level 1 evidence on the survival advantages of brachytherapy, SBRT, or protons over another form of radiation therapy (23). In particular, CHHiP (24) and HYPRO30 trials (25) demonstrated that hypofractionated schemes, exploiting the low α/β ratio of PCa, constitute a valid treatment option for HR patients. However, the number of studies involving extreme hypofractionation is relatively low, and a direct comparison of different hypofractionation schemes is still lacking. Therefore, despite being cited in clinical practice guidelines next to moderate hypofractionation schemes, the current level of evidence is too low to implement extreme hypofractionation as a standard of care. In particular, one of the limits consists in the fact that ultra-hypofractionated regimens in HR PCa lead to a risk of higher toxicity in case of prophylactic whole pelvis radiotherapy (WPRT).

Particle therapy has been gaining interest due to the unique physical and radiobiological properties of protons and other heavy ions, including carbon ions, compared to photons. Specifically, the use of carbon ions as a boost is motivated by their sharper dose gradient that ensures a better OARs sparing and by their high relative biological effectiveness (RBE), with a therapeutic effect up to three times higher with respect to photons and protons (10, 11). In addition, carbon ions might make radioresistant clusters more sensitive to subsequent photon therapy. Such unique physical and biological advantages make them a valuable candidate in the treatment of PCa.

Safety and effectiveness evidence on carbon ions in the treatment of PCa mainly derive from the Japanese experience. The first clinical trial employing CIRT in PCa was activated at the National Institute of Radiological Sciences (NIRS) and dates back to 1994 (26). At the same institute, three additional phase I/II trials over a 13-year time frame and two phase II trials demonstrated the high potential of CIRT in the treatment of PCa. A study by Nomiya et al. (27), aiming at evaluating the feasibility of a 3-week CIRT treatment schedule for PCa, reported G0 and G1 GU acute toxicity events in 10 (22%) and 34 (74%) of patients, respectively, and acute G2 urinary frequency in only two patients (4%). Similar findings were reported by Akakura et al. (12), who analysed the outcomes of a series of 96 patients treated with CIRT +/− ADT in adjuvant or neoadjuvant settings, reported no patients exhibiting G3 or higher acute radiation-induced toxicity. The first prospective observational study conducted outside NIRS was the one by Kawamura et al. (28), which reported low GU and GI toxicities as well as an acceptable biochemical control during the first 5 years following moderately hypofractionated CIRT for localized PCa. These results are in line with those obtained in the present study, with acute GU G0 and G1 toxicity events occurring in 8/15 (53.3%) and 5/15 (33.3%) patients, respectively. Notably, the risk of toxicity in our protocol was slightly higher considering the larger irradiated volume (prostate + pelvis).

No patients experienced biochemical recurrence. However, follow-up is too short to derive robust results, which can be also affected by the fact that some patients are still receiving hormonal therapy. It is expected that these results will be in line with available evidence, highlighting that this treatment modality has an excellent efficacy profile. Indeed, regarding oncological outcomes, the study by Nomiya et al. (27) reported no biochemical failures or distant metastases at last follow-up, with PSA showing a good response in most patients (94%). At 5-year, Akakura et al. (12) reported an overall, cause-specific, clinical recurrence-free, and biochemical recurrence-free survival rates of 87.7, 94.9, 90, 82.6%, respectively. Local control was achieved in all patients but one. Analogously, a study by Kasuya et al. (29), who analysed the treatment outcomes of HR localized PCa treated with CIRT + ADT compared with standard treatment modalities, demonstrated that the association of these two treatments yielded quite favourable treatment outcomes, with biochemical recurrence occurring in 90 out 608 (14.8%) patients, with a median follow-up of 88.4 months. The 5/10-year rates of PCa specific mortality (PCSM) and overall mortality, including PCSM and non-prostate cancer specific mortality, were 1.5 and 5.0%, respectively. Analogously, Kawamura et al. (28) reported a 5-year biochemical relapse-free rate of 92% in the HR group.

The efficacy of dose-escalated EBRT to the prostate alone in patients with HR disease might be limited by the increased likelihood of occult pelvic lymph node metastases outside of the radiation field. The advantage of such mixed beam approach is to irradiate the whole pelvis with a prophylactic intent and, at the same time, to escalate the dose to the prostate. This approach is expected to increase locoregional control by eradicating micrometastatic lesions in the pelvis without jeopardising OARs sparing.

As mentioned above, patient- and physician-reported outcomes were overall satisfactory. The only exception was represented by the erectile function, whose worsening was observed at the end of RT and at 12 months. However, these findings need to be interpreted carefully. To start with, it is important to consider that erectile function was assessed only on six patients, as they were the only ones having a follow-up of at least 12 months at the time of the study. Additionally, it should be taken into account that such observed worsening might be the result of cumulative side effects of ADT and RT. More mature results about erectile function and more in general on all the considered patient’s and physician’s reported outcomes will be available after all patients will have completed the hormonal therapy course (i.e., up to 2 years after ADT beginning) and will shed light on the actual impact of ADT on toxicity outcomes.

As stated above, this paper mainly analyses acute toxicity events. The analysis on oncological outcomes and late toxicities will be performed when more mature data will be available and will be object of a separate publication. As of today, no patients experienced biochemical recurrence. However, follow-up is too short to derive robust results, which can be also affected by the fact that some patients are still receiving hormonal therapy. It is expected that these results will be in line with available evidence, highlighting that this treatment modality has an excellent efficacy profile.

This study, which explores the combination of different RT approaches in the treatment of HR PCa, represents a novelty in the modern RT scenario. This experience proved the feasibility of this novel RT workflow, including safe sharing of medical imaging data between centres *via* trusted channels, effective RT plans sum for dosimetric considerations, as well as an acceptable overall treatment duration for the enrolled patients.

However, the study suffers from some limitations. First of all, the accrual was scarce and lower than expected, due to the fact that most patients with HR PCa undergo surgery. This is mainly due to the fact that the indication for surgery moved from low-risk patients, who are candidate for active surveillance according to most recent guidelines, to patients with HR disease. Therefore, the number of patients to carry out the analysis was low and the follow-up was short. However, the absence of severe toxicity encourages further investigations in this setting.

## Conclusions

In conclusion, results from this preliminary analysis demonstrated the overall safety of such combined treatment modality, due to the low incidence of acute GU/GI toxicities and promising QoL scores following CIRT for PCa. These findings confirm the available evidence on CIRT safety, even with larger irradiated volumes. Therefore, available data on efficacy about CIRT in HR setting seem encouraging and could confirm a new role of carbon ions in this clinical setting.

## Data Availability Statement

The raw data supporting the conclusions of this article will be made available by the authors, without undue reservation.

## Ethics Statement

The studies involving human participants were reviewed and approved by Ethics Committee (R86/14-IEO98) of the European Institute of Oncology (IEO). The patients/participants provided their written informed consent to participate in this study.

## Author Contributions

Conceptualization, GM, BV, RV, MC, RR, FV, TG, FC, EO, BJ-F, and RO. Methodology, GM, BV, FP, SC, GC, DZ, MA, SR, and SM. Validation, GM, MP, MZ, and FP. Formal analysis, FB and SG. Investigation, GM, BV, RV, MC, RR, FV, TG, EO, BJ-F, and RO. Data curation, GM, MP MZ, and FP. Writing—original draft preparation, GM, MP, MZ, FP, FB, and SG. Writing—review and editing, BV, SC, GC, DZ, MA, CF, SR, SM, MC, RR, FV, TG, BA, RV, OC, FC, EO, BJ-F, and RO. Supervision, BJ-F. Project administration, RO. Funding acquisition, RO. All authors contributed to the article and approved the submitted version.

## Funding

FP was partially supported by Associazione Italiana per la Ricerca sul Cancro (AIRC), project IG-14300 “Carbon ions boost followed by pelvic photon intensity modulated radiotherapy for high-risk prostate cancer,” registered at ClinicalTrials.gov (NCT02672449), approved by IEO R86/14- IEO 98. MZ received a research fellowship by the European Institute of Oncology-Cardiologic Center Monzino Foundation (FIEO-CCM), with a project entitled “Proton therapy vs photon-based IMRT for parotid gland tumors: A model based approach with Normal Tissue Complication Probability (NTCP)” outside the current study. SV and FB are PhD students within the European School of Molecular Medicine (SEMM), Milan, Italy. This work was also partially supported by the Italian Ministry of Health with Ricerca Corrente and 5x1000 funds. The sponsors did not play any role in the study design, collection, analysis, and interpretation of data, nor in the writing of the manuscript, nor in the decision to submit the manuscript for publication.

## Conflict of Interest

The authors declare that the research was conducted in the absence of any commercial or financial relationships that could be construed as a potential conflict of interest.

## Publisher’s Note

All claims expressed in this article are solely those of the authors and do not necessarily represent those of their affiliated organizations, or those of the publisher, the editors and the reviewers. Any product that may be evaluated in this article, or claim that may be made by its manufacturer, is not guaranteed or endorsed by the publisher.

## Abbreviations

ADT, androgen deprivation therapy; AIRC, Associazione Italiana per la Ricerca sul Cancro (Italian Association for Cancer Research); CIRT, carbon ion radiotherapy ; CNAO, Centro Nazionale di Adroterapia Oncologica (National Center of Oncological Hadrontherapy); CT, computed tomography ; CTV, clinical target volume ; DIL, dominant intraprostatic lesion; EBRT, external beam radiotherapy; ECOG, Eastern Cooperative Oncology Group; EORTC, European Organisation for Research and Treatment of Cancer; G, grade; GG, grade group; GI, gastrointestinal; GS, Gleason score; GU, genitourinary; HR, high risk; IIEF-15, International Index of Erectile Function; IEO, Istituto Europeo di Oncologia (European Institute of Oncology); IMRT, intensity-modulated radiotherapy; INT, Istituto Nazionale dei Tumori (National Cancer Institute); iPSA, initial PSA; IPSS, International Prostatic Symptoms Score; IQR, interquartile range; LET, linear energy transfer; NCCN, National Comprehensive Cancer Network; NIRS, National Institute of Radiological Sciences; PCa, prostate cancer; PCSM, prostate cancer specific mortality; PSA, prostate-specific antigen ; PTV, planning target volume ; QLQ-C30, Quality of Life Questionnaire-Core 30; QLQ-PR25, Quality of Life Questionnaire PRostate specific; QoL, quality of life; RBE, relative biological effectiveness; RT, radiotherapy; RTOG, Radiation Therapy Oncology Group; SBRT, stereotactic body radiotherapy; T, tumour; WPRT, whole pelvis radiotherapy.
