# Supplementary material for: Mixed-Beam Approach for High-Risk Prostate Cancer Carbon-Ion Boost Followed by Photon Intensity-Modulated Radiotherapy: Preliminary Results of Phase II Trial AIRC-IG-14300
Source: Front Oncol. 2021 Nov 17;11:778729. doi: 10.3389/fonc.2021.778729 (PMC8635961; doi:10.3389/fonc.2021.778729)
Supplement: Supplementary file 1 [file DataSheet_1.docx]

**Figure S1.** The iter of patients enrolled in the trial


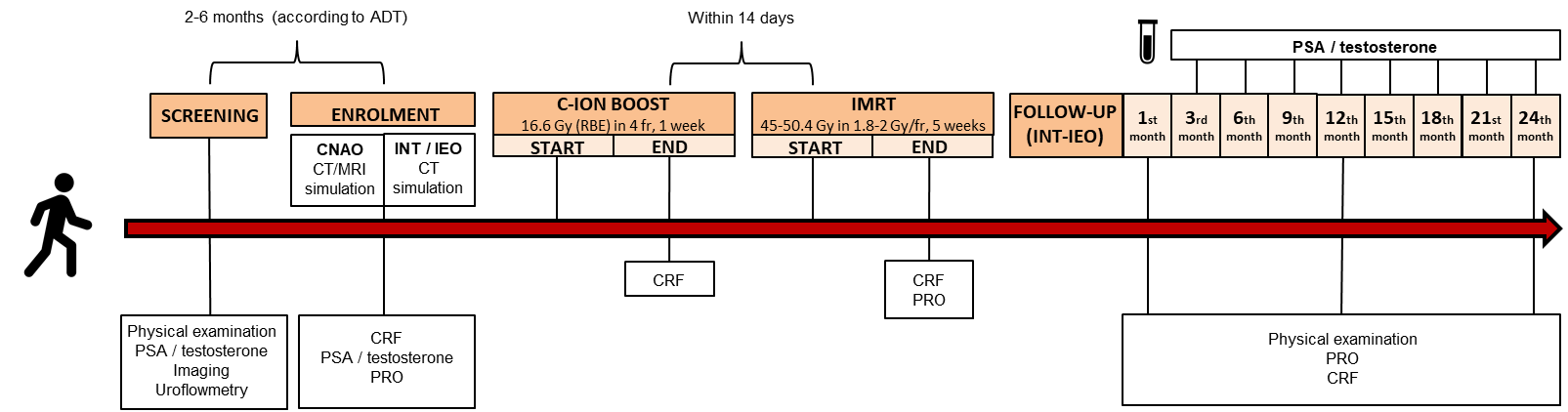


**Abbreviations –** **ADT:** Androgen Deprivation Therapy; **C-ION:** Carbon Ions; **CNAO:** Centro Nazionale di Adroterapia Oncologica (National Centre of Oncological Hadrontherapy); **CRF:** Case Report Form; **CT:** Computed Tomography; **fr:** fraction; **Gy:** Gray; **IEO:** Istituto Europeo di Oncologia (European Institute of Oncology); **IMRT:** Intensity-Modulated Radiotherapy; **INT:** Istituto Nazionale dei Tumori (National Cancer Institute); **MRI:** Magnetic Resonance Imaging; **PRO:** Patient- and Physician- Reported Outcome; **PSA:** Prostate-Specific Antigen; **RBE:** Relative Biological Effectiveness;
